# Variations of telencephalic development that paved the way for neocortical evolution

**DOI:** 10.1016/j.pneurobio.2020.101865

**Published:** 2020-11

**Authors:** Fernando García-Moreno, Zoltán Molnár

**Affiliations:** a Achucarro Basque Center for Neuroscience, Scientific Park of the University of the Basque Country (UPV/EHU), 48940, Leioa, Spain; b IKERBASQUE Foundation, María Díaz de Haro 3, 6th Floor, 48013, Bilbao, Spain; c Department of Neuroscience, Faculty of Medicine and Odontology, UPV/EHU, Barrio Sarriena s/n, 48940, Leioa, Bizkaia, Spain; d Department of Physiology, Anatomy and Genetics, Sherrington Building, University of Oxford, Oxford, OX1 3QX, UK

**Keywords:** Homology, Pallium, Tangential and radial migration, Neocortex, Birds, Reptiles, Vertebrates, Neural progenitors, Hyperpallium, Amygdala

## Abstract

Charles Darwin stated, “community in embryonic structure reveals community of descent”. Thus, to understand how the neocortex emerged during mammalian evolution we need to understand the evolution of the development of the pallium, the source of the neocortex. In this article, we review the variations in the development of the pallium that enabled the production of the six-layered neocortex. We propose that an accumulation of subtle modifications from very early brain development accounted for the diversification of vertebrate pallia and the origin of the neocortex. Initially, faint differences of expression of secretable morphogens promote a wide variety in the proportions and organization of sectors of the early pallium in different vertebrates. It prompted different sectors to host varied progenitors and distinct germinative zones. These cells and germinative compartments generate diverse neuronal populations that migrate and mix with each other through radial and tangential migrations in a taxon-specific fashion. Together, these early variations had a profound influence on neurogenetic gradients, lamination, positioning, and connectivity. Gene expression, hodology, and physiological properties of pallial neurons are important features to suggest homologies, but the origin of cells and their developmental trajectory are fundamental to understand evolutionary changes. Our review compares the development of the homologous pallial sectors in sauropsids and mammals, with a particular focus on cell lineage, in search of the key changes that led to the appearance of the mammalian neocortex.

## Highlights

• To reveal how the neocortex emerged during evolution we need to understand the evolution of the development of the pallium.
• The developmental trajectories of cortical cells are fundamental to understand evolutionary changes and homologies.
• Subtle variations from early brain development accounted for the diversification of vertebrate pallia and neocortical origin.

## 1 Introduction

The original definition of homology was very straightforward, two structures were considered homologous when they derived from a common ancestor structure (Darwin, 1859; Nixon and Carpenter, 2012; Owen and Cooper, 1843). In simple terms, the human arm (i.e., arm, forearm, and hand) and the whale fin were considered homologous as forelimbs, as the common ancestor to these two species already possessed a forelimb. They are not homologous to each other as either arms or fins per se. The original definition for *homology* did not assume any similarity in function, leaving the history of physiological function to be described by *analogies* (for a comprehensive review see (Ochoterena et al., 2019)). As stated by Richard Owen, founder of modern comparative anatomy, homology implies “*same organ in different animals under every variety of form and function*” (Owen and Cooper, 1843; De Beer Sir, 1899-1972, 1971De Beer, 1971De Beer, 1971De Beer Sir, 1899-1972, 1971De Beer, 1971De Beer Sir, 1899-1972, 1971). In the example above, the human arm and the fin of a whale are homologous, as the distant common ancestor of them possessed a structurally equivalent forelimb, and the two structures develop from a similar sector of the developing embryo, i.e., cells are produced from progenitors that are located in homologous sectors of the tissue (forelimb bud) and the initial lineage is comparable, although the functions of these limbs are very different. There are some changes in the production and maintenance in the elements of the human arm and the fin of the whale. Detailed developmental studies reveal the mechanisms that diverged and explain these differences. It is all very straightforward. However, when it comes to the vertebrate brain, its status as one of the most complex biological structures means that untangling its evolution is far more complicated than that of the vertebrate forelimb. We are only at the beginning of understanding of the diverging developmental mechanism that produce different brains.

How can *we* guarantee a body part, such as a given brain structure, was present in extinct species? For the many non-fossilizable features including the brain, its ancient presence can be traced back in evolutionary terms by studying its developmental formation. The developing program not only gives insight into the path for the generation of an organ, but also keeps a record of its evolutionary history (Garstang, 1922; Holland, 2016). Homology implies the sharing of origin and lineage in the developing embryos. Thus, comparing how the brain develops in different species will provide definitive answers to the unsolved questions on brain homology. No assertion is better than Källen´s original “*the longer the developments of two nuclei are similar, the stricter is the homology between the nuclei*” (Källén, 1951). Following this, neocortical features may show analogy to a varied range of other vertebrate brain features. Only the structures generated equivalently to the neocortex, if any, will be true homologues (Wagner, 1996). The same concept applies to the experimentalist’s bench. We can directly correlate sectors of the neuroepithelium that will give rise to specific cell groups, although these groups may change proportion and position during phylogeny by means of alterations of neurogenic programs -i.e., progenitors of these identical sectors of the neuroepithelium might change their developmental behavior, cell cycle dynamics, or lineage.

The purpose of our review is to explore the evolutionary path of the forebrain of amniotes (reptiles, birds and mammals), by comparing the developmental themes and variations that led to the building of the vertebrate brain, with a detailed attention to the cerebral cortex. We shall review recent developmental studies exploring all aspects of brain development in amniotes, from progenitor behavior to neuronal migration, differentiation and wiring. We shall also review the recent literature of single cell transcriptomics in reptiles and mammals in search of cell-type specific markers. We propose that variations from early neurogenic stages had a cumulative influence similar to a snowball effect, and were the main cause of vertebrate brain diversification. Starting by early differences in the secretion of morphogenetic cues led to divergences in the areal patterning of the pallium. As a consequence, the speed of the mitotic cycles increased in different areas of the pallium in different species, which in addition boosted an increase of progenitor cell diversity and compartmentalization. Cell variety increased by segregating neurogenesis in space and time and with the evolution of transcriptional networks that relate to each other in a novel fashion. The novel progenitor cells produced more numerous and more diverse neurons, which located and connected in different manners partly due to additional contributions of novel tangential migrations of glutamatergic neuronal populations. Supported by current EvoDevo research, in particular with comparative cell-lineage analysis studies in mammals, reptiles and birds, we aim to elucidate the developmental variations that likely had an impact on brain evolution, with a specific focus on the neocortex - the brain assembly that most defines mammalian species.

## 2 Variations in pallial patterning in the early vertebrate embryos

The development of the entire nervous system starts with the induction of ectoderm by mesoderm that will form the neural plate, which subsequently folds and fuses dorsally to form the neural tube. At the closure of the neural tube and before the first neurons are born, the vertebrate brain shows a widely conserved morphogenetic and structural *bauplan* (Nieuwenhuys and Puelles, 2015). According to the laws of embryology (Abzhanov, 2013; Von Baer, 1828) which relates the embryonic development of a taxon to its phylogeny, this plan is likely to be very similar to the embryonic brain of the last common ancestor of amniote species. At the rostral region of the neural tube, the telencephalon gives rise to the sensory processing areas of all vertebrates, including the mammalian neocortex- and shows two major divisions: the subpallium (SPall) that generates mostly GABAergic neurons, and the pallium (Pall), which is the source of telencephalic glutamatergic neurons (Anderson et al., 1999; De Carlos et al., 1996; Hevner et al., 2006; Tamamaki et al., 2003) Based on conserved gene expression patterns of a few master developmental genes and transcription factors (Emx1, Pax6, Dlx1-2, Nkx2.1, among many others), the same subdivisions of both Pall and SPall are identified in the embryos of all living amniotes (Puelles et al., 2000; Smith-Fernandez et al., 1998). This supports the conservation of this telencephalic organization down to the last common ancestor. The raw material which can lead to the generation of the diversified amniote brains is already present in the primordial telencephalon of all living and extinct amniotes at this early stage.

**Fig. 1:**
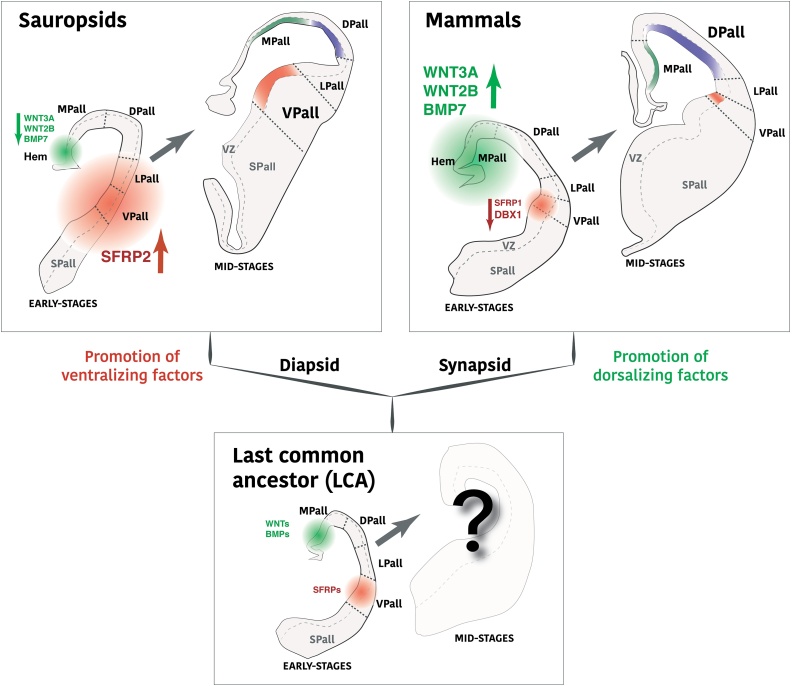
Diverging areal patterning of the pallium in amniote evolution. Outline of brain coronal sections of different vertebrate groups and embryonic stages. For each vertebrate group (sauropsids, mammals and a hypothetical last common ancestor to all amniotes), the left outline represents the disposition of the pallial domains, as well as the suggested intensity signal of areal secreted factors. Dorsalizing factors are represented in green, more preeminent in mammalian brains; ventralizing factors are shown in red, more dominant in sauropsids. The right outline for each vertebrate group represents the extension of each pallial domain at mid-neurogenesis.

Intriguingly, it is also at this earliest stage when the first variations become apparent to the common telencephalic theme, even before the generation of the first neurons. In spite of its highly conserved early patterns, the regional organization of the pallium shows subtle differences in the levels of expression of crucial homeobox transcription factors and soluble patterning factors. Some of these signals originate from outside of the nervous system, some from the edges of telencephalic vesicle (Grove et al., 1998; Rubenstein et al., 1999). As a consequence, the pallial subdomains differ in their size and relative importance in early telencephala. For example, the sauropsid telencephalon presents an increased ventral domain (the ventral pallium, VPall) (Medina and Abellán, 2009) compared to the mammalian pallium where the dorsal sector (dorsal pallium DPall) dominates (Fig. 1). These two domains differ in their expression of *Emx1*, which is absent in VPall but present in DPall (Puelles et al., 2000). What variations made them grow differentially across vertebrates? Only a handful of morphogenetic cues are known to differ across species that could account for these variances. For instance, the mammalian VPall precursors express the transcription factor Dbx1 (Bielle et al., 2005; Luis Puelles et al., 2016a); in contrast to sauropsid VPall cells that do not (Bielle et al., 2005; García-Moreno et al., 2018). This transcription factor is known to regulate the type of division in pallial precursor cells, favoring exit from the cell cycle rather than leading to the self-renewal of the precursor by symmetric division (García-Moreno et al., 2018). Accordingly, the lack of Dbx1 expression by sauropsid VPall precursors could account for an increase of VPall germinative size due to increased self-renewal divisions. On the other hand, Dbx1 expression in mammalian VPall could induce a diminished VPall relative size. This diminished VPall might support a relative increase of the dorsal sector, as DPall is the largest mammalian pallial sector.

**Fig. 2:**
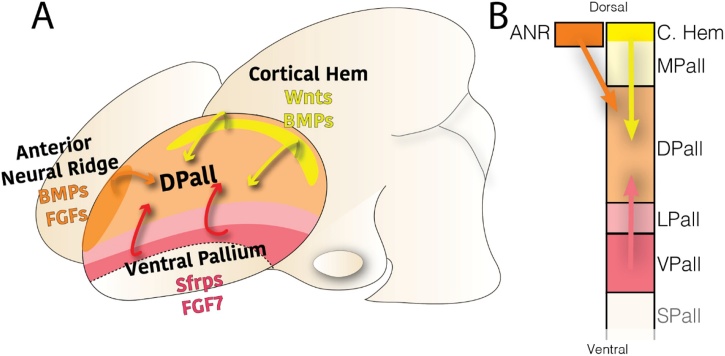
Secreted factors from the edges of telencephalic vesicles affect pallial domain proportions. A. Tridimensional scheme (rostral at the left) of a mammalian early embryonic brain. B. Pallial subdomains in dorsal-top to ventral-bottom disposition. The schemes highlight the location of the main organizers of pallial development and their influence (arrows) over pallial development. The dorsal pallial area receives secreted patterning proteins from all its edges.

Another factor regulating pallial subdomain size across species might be secreted proteins acting from the edges of the pallium (Fig. 1, Fig. 2). Secreted proteins operate in a similar way to transcription factors in that their presence affects subdivision size. The pallial edges are the cortical hem at the medial side (Grove et al., 1998); and the ventral pallium at the lateral side (also called the anti-hem, due to its opposed regulatory effects) (Kawano and Kypta, 2003; Kim et al., 2001). On the medial edge, the cortical hem expresses and secretes a range of morphogenetic factors, such as WNT and BMP proteins, that dorsalize the affected areas (Caronia-brown et al., 2014; Furuta et al., 1997; Fig. 2). For instance, in mouse embryos lacking the downstream target of the Wnt signaling, Sfrp1, the medial pallial domains are reduced in size whereas the DPall is medially expanded (Esteve et al., 2018; Tole and Grove, 2001). In addition, genetic ablation of the cortical hem in *Wnt3a* knock out mice also leads to a decrease of the caudo-dorsal neocortical epithelium, and an expansion of its ventrolateral portion (Caronia-brown et al., 2014; Caronia-Brown et al., 2016). The VPall, on the other hand, secretes FGF7 and orthologous frizzled-related proteins (Sfrps) 1 or 2, depending on species -Sfrp1 in avian VPall, Sfrp2 in mammalian VPall (Assimacopoulos et al., 2003; Frowein et al., 2001; Rattner et al., 1997)- which induces a ventralized phenotype. Failure in this signaling, or in its downstream pathway led by Pax6, drives the lateral portion of the pallium towards a more dorsal- and neocortical-like phenotype (Assimacopoulos et al., 2003; Ferri and Levitt, 1993). In fact, the transcription factors crucially regulated by WNTs and BMPs, Pax6 and Emx2, exert the dorso-ventral patterning of the pallium, and mice lacking each of them display abnormally developed pallial subdomains (Bishop et al., 2002; Bishop and Goudreau, 2000; Jones et al., 2002; Mallamaci et al., 2000). The typical secreted proteins at both hem and antihem, and other opposing factors, are hypothesized to diverge in their relevance across amniote species (Aboitiz and Zamorano, 2013). The sauropsid early pallium might be highly influenced by a strong signaling from the VPall. As a consequence, the ventral domains of the early pallium are larger with respect to the dorsal domains. This early difference in size of the pallial subdivisions later translates into immense disparities: a large densely-populated dorsal ventricular ridge versus diminished dorsal derivatives (Montiel and Aboitiz, 2015). This is one of the main features of sauropsid pallia. On the other hand, the mammalian early pallium might show a prevalence of dorsalizing factors secreted from the dorsal midline. As a consequence, dorsal domains of the early pallium are relatively larger than the ventral domains. And this variation in pallial size produces the relatively expanded neocortex. The transition and switch between these diverging formations is obvious in the Pax6 cKO (Molnár, 2011). Deletion of Pax6 transformed the early arrangement of the pallial subpallial boundary and cells failed to migrate ventrolaterally and protruded into the lateral ventricle as a large ball, which resembled the sauropsid scenario of the dorsal ventricular ridge (Figs. 4G and 5 G in (Jones et al., 2002)).

The differences in the size of pallial compartments are well established throughout amniote evolution (Puelles et al., 2000). However, it remains unknown whether the factors mentioned above are responsible for the observed changes or are secondary features, with the cellular mechanism explaining these variations also unknown. Most likely, dorsal neural progenitors respond to patterning factors, namely Gli3, Shh, Wnt3a, Wnt2b, Fgf8, Fgf7, Sfrp1, Emx2, Pax6, Ngn2, Pbx, CoupTf1 or Spry (Alfano et al., 2014; Caronia-brown et al., 2014; Faedo et al., 2010; Garda et al., 2002; Garel et al., 2003; Golonzhka et al., 2015; Grove et al., 1998; Machold et al., 2003; O’Leary and Sahara, 2008; Rash and Grove, 2007; Yabut and Pleasure, 2018). These factors control the cellular proliferative profile in different ways across the pallium, as their effect is diffused and graded. Most of these proteins are well conserved among amniotes, and they diversified their function by variations of their levels of expression on each species. This variation was achieved by differential evolution of gene regulatory mechanisms. A high specifity of regulatory elements to discrete embryonic telencephalic domains has been described, a specificity that is rarely seen for gene expression, especially within the cortical primordium (Pattabiraman et al., 2014). Altogether, each pallial progenitor varies in the degree of influence on these patterning factors. Each pallial domain differs in the impact they have on neuronal production output, which explains the observed differences in pallial derivative size across vertebrate taxa (Fig. 1, Fig. 2).

## 3 Neural progenitor differences

The neural tube has an inner surface, named the ventricular zone that is adjacent to the ventricle. The outer surface is covered with a basal membrane and the pia mater; therefore, it is called the pial surface. In cell biological terms, the pial surface is called basal and the luminal surface apical, as for all other epithelial tissue. Cell divisions occur at the ventricular or apical surface of the neuroepithelium. Germinative zones in the pallium of amniote brains also display strong conservation prior neurogenesis. However, as soon as neurogenesis starts, pallial neural stem cells in amniotes show regional variations and a general trend to diversify. A diverse and densely-populated embryonic progenitor pool feeds the increased demand for neuronal number of the embryonic brain, as a more elaborated hardware is required for complex information processing (Striedter, 2004). Large brains, populated with more diverse neuronal populations that could assemble into complex circuits enabled greater behavioral complexity with advantages that define the amniote taxon, and this could only be achieved by diversification and amplification of output from pallial progenitors. In addition, neural stem cells also diverged across amniote species. We postulate that these divergences were likely initiated by disparities in the graded expression of morphogenic factors from the telencephalic signaling centers. We propose here two major developmental traits as possible sources of progenitor diversity and divergence: ventricular detachment (Smart et al., 2002) and heterochronic delay of cell-dynamics. In fact, these two processes have been proposed to be well interrelated (Charvet and Striedter, 2011).

### 3.1 Ventricular detachment of pallial progenitors

**Fig. 3:**
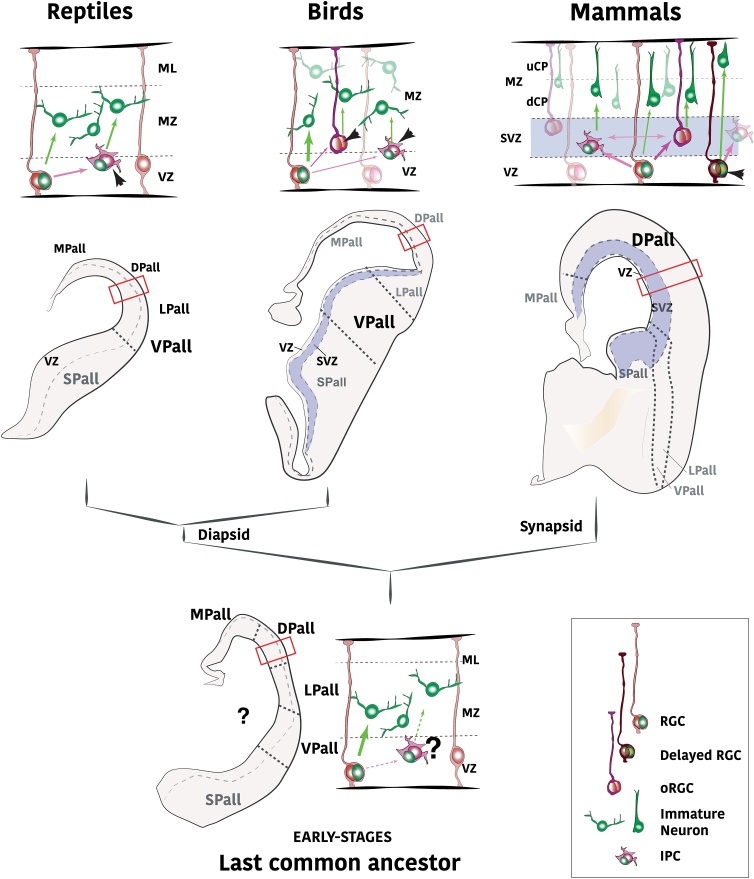
Diversification of progenitor pools in dorsal pallial evolution. Top row: the developing dorsal pallial neuroepithelium and their cell constituents in reptiles, birds and mammals. Middle row: scheme of the location and size of the major germinative zones: ventricular (VZ) and subventricular zones (SVZ). The examples represent gecko, chick and human early telencephala in coronal section. At the bottom, equivalent schemes of pallial neuroepithelium and coronal section of the telencephalon of the hypothetical last common ancestor to all amniotes. dCP, deeper cortical plate; IPC, intermediate precursor cell; ML, molecular layer; MZ, mantle zone; oRGC, outer radial glial cell; RGC, radial glial cell; uCP, upper cortical plate.

Stereotypical neural stem cells are located in the germinative zone, within the ventricular zone (VZ) (Bystron et al., 2008). The apical end feet of the radial glial VZ progenitors are fixed to the ventricular surface, and depend on this attachment for their appropriate cell division. This population acts as a starting point for neuronal production, and, given that the ventricular surface is finite, the population size is spatially restricted to a maximum number of dividing cells. To overcome this restriction, a similar strategy is observed in densely populated pallial regions across vertebrates (Abdel-Mannan et al., 2008; Aboitiz, 2011; Fish et al., 2008). During embryonic development, some radial glial progenitors release their attachment to the ventricular surface, and their nuclei move basally towards the subventricular zone (SVZ) and divide for a further fixed restricted number of cycles (Fig. 3). This way, a given ventricular surface area possesses two mitotic compartments (VZ and SVZ), and it is capable of producing a higher number of neurons in the same restricted area (Cheung et al., 2010; Fish et al., 2008; Noctor et al., 2004; Smart et al., 2002; 1985). An organized SVZ can be found in both leading domains of the vertebrate pallium: the mammalian dorsal pallium (García-Moreno et al., 2012; Smart et al., 2002) and the sauropsid ventral pallium (García-Moreno et al., 2018; Nomura et al., 2016), but also in the dorsal pallium of selected avian species (Charvet and Striedter, 2011; Striedter and Charvet, 2009). In the rest of the brain, the SVZ is not structured as a segregated and organized compartment of cycling neural progenitors located at a similar distance from the ventricular surface, which raises the question of how it appeared in evolution. The absence of a reptilian dorsal SVZ (Martínez-Cerdeño et al., 2006; Molnár et al., 2006) suggests that both mammalian and avian SVZs evolved separately. Therefore, the molecular mechanism that led to the doubling of the proliferative compartments might be different in the two main amniote taxa.

Some data point to a conserved mechanism of detached progenitor production in birds and mammals. Precursors detaching from the ventricular surface are originally termed outer radial glial cells, or basal RGs since they are closer to the basal surface of the neuroepithelium (bRGs or oRGS)(Fietz et al., 2010; Hansen et al., 2010; Nomura et al., 2016), which together with intermediate progenitors (IPs, discussed below), populate the SVZ and promote an increase in the neurogenic output. bRGs have been found both in mammalian and sauropsid embryos (Fietz et al., 2010; Hansen et al., 2010; Nomura et al., 2016), at the most active developmental pallial regions. Outer radial glia (oRG), also called basal radial glia (bRGs), maintain most of the features that define a radial glial progenitor: cell polarity, nuclear movement associated with mitosis (Ostrem et al., 2014), contact with the pial surface, and expression of Pax6 and Sox2. As such, whereas IPCs could be somehow classified as slow-progressing neuroblasts, bRGs are dislocated stem cells whose main differential feature is the detachment from the ventricular lumen. Most likely, *Shh* signaling plays a role in the increased VZ proliferation and the formation of the bRG population (Wang et al., 2016). It suggests that this mechanism to increase neuronal production predated the amniote split and was already present in the embryonic pallium of the last common ancestor.

### 3.2 Heterochronic delay of cell-cycle exit

A change in the timing of cell cycle progression, which affects a subset of progenitors, is a heterochronic variation of the cell cycle. An additional hypothesis that aims to explain the diversification of neural progenitors in amniote evolution focuses on the heterochrony of cell division dynamics. Accordingly, a delayed cell cycle exit -at several steps of the neural stem cell lifespan- was responsible for the origin of the SVZ (Charvet and Striedter, 2011). This cell cycle-exit delay could have affected a subset of newly born cells already committed to the neuronal fate. After division at the VZ, a subset of neuroblasts show features of early immature neurons: they are attached neither to pial nor ventricular surfaces and initiate radial displacement towards the mantle zone (Englund et al., 2005; Vasistha et al., 2015). But as a novelty, these cells do not leave the cell cycle and remain cycling for some hours or days. In the case of the mammalian dorsal pallium, the delay in cell cycle exit may happen on Tbr2-expressing neuroblasts (Fig. 3). T-box brain protein 2 (Tbr2), also known as Eomesodermin, is a transcription factor that drives the differentiation of glutamatergic neurons in the forebrain (Hevner et al., 2006). Radial migration of these immature neuroblasts arrests in the SVZ, and the cells remain cycling for a set number of times, multiplying the neurogenic output (Arnold et al., 2008; Sessa et al., 2010; Vasistha et al., 2015). These still-cycling early-neuroblasts are considered to be intermediate progenitor cells (IPs) (Fig. 3). In the sauropsid ventral pallium, a similar mechanism could be involved, but the delayed cell cycle exit is still a matter of controversy when it comes to Tbr2 expressing neuroblasts (Martínez-Cerdeño et al., 2016; Nomura et al., 2016). Although Tbr2 cells are observed in close proximity to the ventricular area, it is not clear whether these maintain the transitory capacity to divide. Given the high conservation of the Tbr2 protein (*Ensembl* predicts around 80% between mouse and chick), the difference in proliferative capacity of Tbr2-expressing cells may be related to changes independent with this transcription factor. Despite Tbr2 differences, there is, however, a well-organized secondary proliferative layer at the SVZ of specific avian pallia (Charvet and Striedter, 2011; García-Moreno et al., 2018; Nomura et al., 2016; Striedter and Charvet, 2009). Thus, a heterochronic delay may also take place on avian IPs, but this might occur in another cellular population with currently unknown features, and not in Tbr2-expressing early neuroblasts (Cheung et al., 2010).

A second heterochronic delay of cell dynamics acts on some dorsal pallial RG progenitors of the mammalian brain. Whereas a cell cycle exit delay which generates IPs may occur on newly generated immature neurons, an equivalent delay may happen on a subset of radial glial progenitors of the DPall (Franco et al., 2012; García-Moreno and Molnár, 2015) (But see opposite interpretations in Guo et al., 2013). This population does not become neurogenic when most RG cells are. The neuronal progeny output of these progenitors differs from the others due the temporal restriction associated with the neuronal lineage (Franco and Müller, 2013), and so these delayed RG comprise a segregated RG type. Because *neurogenic time* is the main factor providing these delayed progenitors with a differential lineage -and therefore have a major evolutionary impact- these will be discussed in the section examining time-restricted lineages. However, they constitute another example linking progenitor variety to increased neuronal outcome and diversity.

One way in which brain diversity is achieved, thus, is through the differential amplification and diversification of the several stem cell pools. The mammalian dorsal pallium reached the highest complexity by diversifying RGs at the VZ, and maximizing both IPs and bRGs at the SVZ (Fig. 3). The avian ventral pallium, on the other side, alternatively enlarged its neurogenic output by promotion of an organized SVZ following yet unknown mechanisms (Charvet and Striedter, 2011). These vertebrate neural stem cell pools provided a great boost to neuronal numbers of particular pallial areas, propelled their evolution, and augmented a behavioral repertoire that was selected for in the population.

This is the miracle of evolution that these alterations in neurogenesis produced sets of neurons that assembled into circuits that produced some form of sensory, motor or adaptive benefit to the adult individual. Changes in properties and arrangements of progenitors at different regions of the telencephalon produced variation that was screened for by selectional advantages and functional benefits for millions of years. The types of mitoses (self-renewing versus direct neurogenic) have also evolved to contribute to the enlargement of certain pallial areas (Cárdenas et al., 2018). But there is another factor that links the differential evolution of pallial areas at a cellular level: tangential movements of immature neurons.

## 4 Tangential migrations contributing to pallial development

The differential compartmentalization of neurogenesis in each subdomain was not isolated from the rest of the pallium. Each pallial domain influences the others, either by means of diffusible morphogenic factors or by tangential movements of cells. Those tangential migrations -cellular movements from one brain compartment to another- and their developmental contributions evolved independently in both amniote taxa. Adult vertebrate brains reflect the differential impact of tangential migration on their morphology and function (García-Moreno and Molnár, 2020). In the case of the pallium, the tangentially migratory, subpallial GABAergic neurons are present in all vertebrate species studied so far, and their migration through the pallium is conserved throughout the entire vertebrate radiation (Cobos et al., 2001; Métin et al., 2007; Carrera et al., 2008; Moreno et al., 2008, 2010; García-Moreno et al., 2018). On the other hand, glutamatergic intrapallial tangential migrations evolved independently (García-Moreno et al., 2018) and it is thought that this difference had a wider impact on neocortical evolution.

**Fig. 4:**
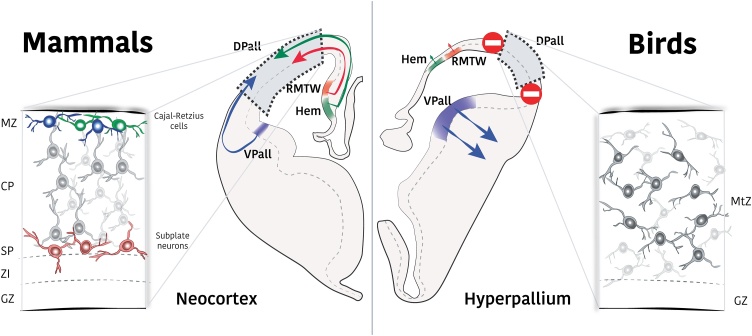
Glutamatergic tangential contributions to neocortical evolution. Left panel: in mammalian brains, several populations of glutamatergic neurons reach the developing DPall by tangential migration. Neurons born at the cortical hem, rostral medial telencephalic wall (RMTW, depicted in the coronal slide, but actually present at more anterior telencephalic levels) and VPall migrate tangentially towards the DPall. The developing neocortex contains Cajal-Retzius cells, subplate neurons and transient pyramidal neurons (black), which play essential roles in neocortical formation. Right panel: The homologous pallial regions of the avian telencephalon do not give rise to tangential migrations. The chick DPall does not receive external glutamatergic contributions. As a consequence, crucial populations for neocortical development are lacking in the developing avian hyperpallium, whose glutamatergic neurons only derive from DPall progenitors. CP: cortical plate; GZ: germinative zone; IZ: intermediate zone; MtZ: mantle zone; MZ: marginal zone; SP: subplate layer.

Some neuronal populations move tangentially within the developing pallium of mammals. These cells leave the pallial domain where they are generated, move along the surface and reach and settle in a new pallial area. All four pallial subdomains send and receive tangentially migrating cells, which become glutamate-releasing neurons (Mattar et al., 2008, 2004). Neurons from the cortical hem (in the medial pallium) and ventral pallium migrate early in the development towards the DPall neuroepithelium becoming Cajal-Retzius cells and subplate neurons, respectively (Fig. 4) (Bielle et al., 2005; Ceci et al., 2012; García-Moreno et al., 2008, 2007). Once settled in DPall, these two populations exert essential instructions for cortical development (Barber and Pierani, 2016), such as leading the inside-out gradient of neurogenesis, maintaining the radial glial palisade, and modulating thalamo-cortical early wiring (Hoerder-Suabedissen and Molnár, 2015; Katsuyama and Terashima, 2009; Soriano and Del Río, 2005). Abnormal development of these crucial neurons results in the failure of corticogenesis at structural and functional levels.

The existence of these early tangential migratory streams of neurons was investigated in the embryonic chick brain (García-Moreno et al., 2018). *in vivo* full-lineage tracing of the chick cortical hem and VPall revealed a total lack of tangential migration of glutamatergic neurons towards the DPall, contrary to what is observed in mice. The cells originating in these subdomains did not migrate tangentially and stayed within the radial domains of their origins, i.e., the hippocampal area for the cortical hem-generated cells, and the nidopallium for the VPall-generated cells. Therefore, the avian DPall, called hyperpallium, follows a developing program without tangential arrivals from other pallial sources (Fig. 4). As a main consequence, the corticogenic instructions provided in mammals by Cajal-Retzius cells and subplate neurons are missing in birds (García-Moreno et al., 2018). This very early difference might be the reason behind the laminar and columnar organization of mammalian neocortex and the nuclear arrangement within the avian pallium.

It is currently unknown whether reptilian embryonic pallium displays or lacks tangential migrations equivalent to those observed in mammalian brains, but not in chick. We propose a parsimonious scenario in which glutamatergic tangential migration is absent in the reptilian pallium, homologous to birds. The development of these species would likely resemble that of the last common ancestor of amniotes, lacking intrapallial tangential migrations of glutamatergic neurons. We hypothesize that early during the independent evolution of mammals, some genetic changes enabled pallial tangential migrations. These changes most likely occurred as a byproduct of earlier developing divergences such as early pallial patterning and signaling or proliferation dynamics. It remains unsolved whether these potential changes happened in the developmental program of the migratory cells themselves –which would have *de novo* learned to invade the adjacent DPall-, or at the recipient area for these cells, the DPall -which became permissive and/or attractive to cellular invasions-, or both. Either way, new cells arrived in the ancient mammalian DPall and triggered a sequence of developmental events that enabled the appearance of the radially arranged mammalian neocortex. These are key developmental events that are still preserved and present during mammalian cortical development, reflecting the variation of development that produced an altered arrangement that had selectional advantage.

It has been proposed that the lateral and/or ventral pallium contributes neurons to the dorsal cortex. These suggestions are based on gene expression studies of selected markers (Nurr1/ Nr4a2), (L. Puelles et al., 2016c)) and gene expression indicated in reporter gene expressing mice (Dbx-1 lacz, (Luis Puelles et al., 2016a, 2016b; Teissier et al., 2010)). However, lineage tracing studies failed to reveal tangential migration from these sources to the dorsal cortex in mice and chick to date (Bruguier et al., 2020; García-Moreno et al., 2018; Rueda-Alaña et al., 2018). In both amniote groups tested, neurons from lateral and ventral pallia migrated in an equivalent fashion, to form the radial derivatives. In mice, the cell lineage covered the claustrum, insular cortex, dorsal endopiriform nucleus, piriform cortex and amygdala, following the bent course of radial glial processes in the ventral migratory stream (Puelles et al., 2016a, 2016b; Rueda-Alaña et al., 2018). In birds, the lineage of the lateral pallial settled the mesopallium (homologue of the claustro-insular complex) and that of the ventral pallium migrated radially towards the entire nidopallium (homologue of olfactory cortex and amygdala)((García-Moreno et al., 2018) and García-Moreno unpublished results). In reptiles, recent evidence locates the homologue of the claustrum at the most anterior region of the dorsal ventricular ridge, a position consistent with its lateral pallial developmental origin (Norimoto et al., 2020). This novel data suggests a deep conservation of the radially-exclusive development of lateral ventral pallia in amniotes.

The mammalian novelty of these early and later migratory patterns of glutamatergic neurons is in contrast with the highly conserved tangential migratory patterns of GABAergic neurons in sauropsids and all mammals from pallidum (Carrera et al., 2008; Cobos et al., 2001; Metin et al., 2007; Moreno et al., 2008).

## 5 Timing of neuronal generation in the pallium

A key characteristic of amniote pallial evolution is the significant trend to produce more and larger variety of numerous as a result of specialization of progenitors and compartmentalization of proliferative zones. Segregating progenitors in space and time produces differential transcription factor combinatorial codes that produce a larger variety of neurons (Englund et al., 2005). Specifically, *when* during neurogenesis a neuron is born dictates: (1) where in the mature pallium that neuron will be located, and (2) what type of neuron will it become. Evolution tinkered with these two factors, and neurogenic time ended up as one of the most relevant evolutionary drivers and modulators of the structure and function of the mature pallium.

**Fig. 5:**
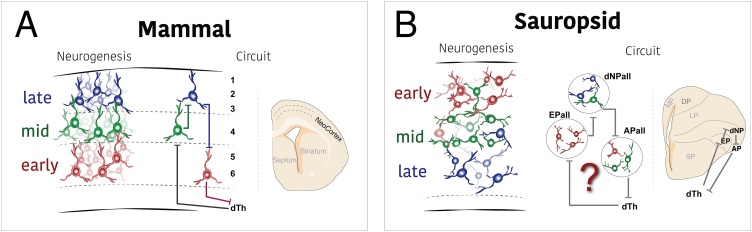
Timing of neurogenesis and circuit formation. A. In mammals, the location of cortical neurons in each layer is determined by their birthdate. As a consequence, the internal circuitry of the cortex is highly dependent on birthdate. B. In sauropsids, the location of cells in the postmitotic zone of the neuroepithelium is opposite to the mammalian case. Although an equivalent internal circuitry is known, we lack information on how the development of the neurons impacted on the circuit function. The example represents the coronal section of the chick and its intra-pallial high order sensory circuit. APall, arcopallium; dNPall, dorsal nidopallium; dTH, dorsal thalamus; EPall, entopallium.

Newborn neurons leave the vicinity of the ventricular area of the neural tube and move towards the mantle zone where they continue to differentiate. In most brain regions and vertebrate taxa, the newly arrived neurons take up a position beneath the previously-born neurons that are already present in the mantle zone (Striedter, 2004). As a consequence, the mature brain displays the earliest-born neurons next to the pial surface, and the latest-born populations in close proximity to the ventricular surface (for instance the pallium of birds (Tsai et al., 1981) or the mammalian diencephalon (Altman and Bayer, 1978a, 1978b)). This positioning order follows a gradient, termed the neurogenic *outside-in* pattern of neurogenesis. It is remarkable that this pattern is inverted in the mammalian dorsal pallium, which is formed based on an *inside-out* gradient of neurogenesis (Angevine and Sidman, 1961). In the embryonic neocortex newborn neurons have tight associations to RGC fibers, and migrate among previously-born neurons to reach a more superficial position above them (Rakic, 1971). This way, the latest-born neurons climb up to the meningeal surface, whereas the oldest populations locate in deeper cortical layers near the wall of the lateral ventricle (Fig. 5). It might have been an advantage to introduce two relatively mature and consistant cell layers. One lies below the cortical plate, between the germinal zone of the cortex and the immature cortical plate, and another one locates above the cortical plate. The layer between the germinal zone and the cortical plate is the subplate layer (Kostovic and Rakic, 1990). The subplate provides a stable platform on which the afferent connections can accumulate while the distances are minimal and while the cortex is being constructed in an inside-first outside-last manner (Allendoerfer, 1994; Kanold and Luhmann, 2010).

Cajal-Retzius cells in the marginal zone are essential regulators of the radial migration that leads to this inside-out gradient. This link is another example of brain evolution enabled by a change in the sequence of events. Novel tangential migration of Cajal-Retzius cells modified the mammalian dorsal pallium *per se*, but it also enabled an inverted pattern of neurogenesis. This is in opposition to sauropsid pallia, which do not display Cajal-Retzius invasion and thus do not invert the neurogenetic gradient (García-Moreno et al., 2018).

These two opposed neurogenic gradients, inside-out in mammals and outside-in in sauropsids, lead to inverted pallial mantle zones in mammals versus sauropsids. This inversion had a larger impact accompanied by the great variations in the neurogenic capacity of different sectors of the pallium: in sauropsids, the dorsal pallium hosts relatively fewer neurons compared to mammals, whereas lateral and ventral pallia host relatively more. Therefore, the shift in neurogenic timing impacted pallial functionality. The internal circuitry of the pallium (Dugas-Ford et al., 2012; Yu et al., 2009), the communication with the thalamus (Bielle et al., 2011), and other key factors of pallial function diverge among amniote taxa due to the contrasting location of its diverse neurons. Alterations in neurogenic timing accounts for these divergences. What caused these developmental differences that drove brain evolution?

The capacity to generate these different gradients resides in the ability of neural progenitors to give rise to multiple types of neurons over time. This capacity is restricted over the course of development (Galazo et al., 2016; Suzuki and Hirata, 2014). An early neurogenic progenitor hosts the capacity to generate different neuronal types over the embryonic period, but it loses this capability division after division. Thus, the same progenitor at late stages possess a restricted neurogenic potential, and will only produce the neurons typical of late neurogenic stages (Frantz and McConnell, 1996; McConnell, 1988), although this concept has recently being challenged (Oberst et al., 2019). This sequential fate-restriction acts irreversibly on cortical progenitors. In homotypic cellular transplants, early progenitors grafted into late-developing cortices turn from an earlier to a later neurogenic stage. However, the opposite change does not occur because the late progenitor is irreversibly fate-restricted. In addition, it has recently been shown that neocortical progenitors generate a given type of cortical neuron according to their membrane resting potential, which varies over the developmental time course (Vitali et al., 2018). Due to the inside-out gradient of cortical neurogenesis, the sequential restriction links the type of neuron generated to the layer in which it will settle. During mammalian evolution, a new mode of radial movement enabled the novel outside-in positioning within the neocortex. Mammalian cortical neurons acquired locomotive radial migration, led by a dynamic regulation of Wnt signaling (Nomura et al., 2020). This change in the way newborn neurons move helped produce a novel mammalian dorsal pallium.

This new mammalian order of positioning of the neurons within the DPall provided a substrate for a change of the internal circuitry, which caused a major impact on brain evolution (Fig. 5). Clonally-related sibling neurons tend to form more connections between themselves than non-related ones (He et al., 2015; Yu et al., 2009) possibly because they maintain gap junctions shortly after migration that could synchronise their signaling even before synapse formation. The new distribution of neurons led to the elaboration of the canonical cortical circuit (Bosman and Aboitiz, 2015), with its trisynaptic structure being considered the basic fundamental processing microcircuit of the neocortex and the basis for neocortex computational efficiency (Harris and Shepherd, 2015). Is this crucial neurogenic timing a mammalian novelty or a feature conserved among amniotes?

A fate-restriction of progenitors has been observed in every CNS studied so far. However, it has been suggested that the pallium of birds and reptiles does not follow a *temporal* restriction equivalent to that of the neocortex. Suzuki and colleagues proposed that the restriction of the avian pallium was not temporal, but spatial (Suzuki et al., 2012). Accordingly, different types of neurons were generated at the medial and lateral edges of the pallium, in opposition to the rather homogeneous mammalian neocortical medial-to-lateral domain. Although the authors show that neurons generated at medial and lateral areas of the pallium belong to different classes, this pattern cannot be compared to that of the single domain within the dorsal pallium that generates the neocortex. The avian progenitors studied by Suzuki and colleagues were located in the medial and lateral pallia (Suzuki et al., 2012), and not in the dorsal pallium as required for the comparison to the neocortex. Currently we lack knowledge of whether the progenitors of the sauropsid dorsal pallium produce different neuronal types at various stages of neurogenesis (Fig. 5), but some data points in this direction. First, the reptilian dorsal cortex is generated in an outside-in neurogenic gradient (Bar et al., 2000; Goffinet et al., 1986) (but see (Xi et al., 2008)). And second, a link has been established between the position of the neurons within the three layers of the cortex and their mature connectivity (Laurent et al., 2016; Tosches et al., 2018). Together, it seems that the time when a neuron is generated determines its function even in the reptilian cortices. We propose that temporal restrictions are present in pallial progenitors of all amniotes, although the fine description of these timing events is lacking in sauropsids and surely will reveal differences to the mammalian case.

Being a key regulator of pallial structure and function, evolution altered the sequential fate-restriction of pallial progenitors to further diversify pallial derivatives. Tinkering with the developmental time, a heterochronic delay of cell cycle exit in cortical progenitors impacted on brain evolution. Several subsets of RGC of the mammalian VZ cycle followed different rules to the rest of the RGC population. This way, the delayed cells are not neurogenic at developmental time-points when most radial glia are (García-Moreno and Molnár, 2015). As a consequence, these progenitors produce a segregated neuronal lineage, different to that derived from not-delayed progenitors. Some progenitors give rise to neurons of the whole-depth cortical grey matter, others only form neurons for the upper layers (Fig. 3, upper row), and a few progenitors only contribute to the development of the deep layers (Franco et al., 2012; Guo et al., 2013; Llorca et al., 2019). These heterogeneous populations of VZ progenitors enabled the exploration of building circuits from greater neuronal diversity and helped in the generation of vastly populated cortices, specifically the dense supragranular layers of carnivore and primate mammals (Hutsler et al., 2005). No similar heterochronic delays or heterogeneous progenitor populations have been described in the sauropsid pallium (García-Moreno and Molnár, 2015). This implies that the emerging heterochronic cell-cycle delays in primordial mammalian embryos could have been a crucial step in the formation of the mammalian neocortex. But also, that the evolutionary increase of ventral pallial derivatives in sauropsids was due to independent cellular and molecular mechanisms. Brain evolution implemented independent instructions to generate large, populated and diverse brains.

## 6 Neuronal type specification

Newborn neurons leave the germinative zones and settle the mantle zone following opposite chronological orders depending on the taxon. These young neurons mature and differentiate into one of many neuronal subtypes present across the forebrain (Zeisel et al., 2018). In general terms, the differentiation into a given neuronal type strongly depends on the neurogenic time of birth and the microenvironment of differentiation. This way, an early-born neuron of the mammalian dorsal pallium becomes a deep layer projecting glutamatergic neuron. However, this fate could vary if the immature neuron finds a different cellular environment to that of the deep layers. Therefore, the mechanisms for neuronal specification might have evolved at both levels, the intrinsic program of differentiation dictated by the developmental program and the extrinsic differentiating environment. Heterochronic transplant experiments have not yet been performed in reptilian or avian dorsal pallium, although they could detect potential differences from mammals in these regards.

**Fig. 6:**
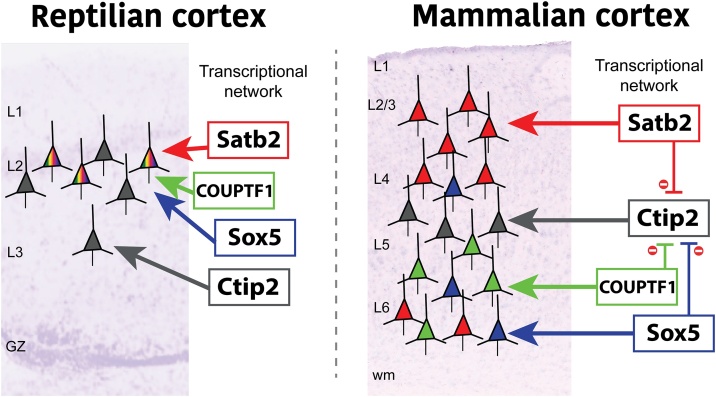
Cross-suppressing transcriptional activity enabled neuronal type diversification in mammals. Left panel: In reptiles and birds, the expression of transcription factors for glutamatergic differentiation of neurons is independent. As a result, many glutamatergic pyramidal neurons express several of these transcription factors. In mammals, right panel, the *cis*-regulatory elements of the same transcription factor genes evolved towards a general cross-suppression of activity. Therefore, mammalian pyramidal neurons express one of these transcription factor determinants. This exclusive expression enabled the individuation of more neuronal types.

The major types of pallial neurons (including the subpallium-originated pallial interneurons) are present across all amniote species studied so far. Among glutamatergic neurons, equivalent types are found in the pallium of chick, gecko, lizard, turtle and mammals. They all share the expression of central transcription factors such as Foxg1, Pax6, Tbr1, RorB, Satb2 and Ctip2 (Briscoe et al., 2018; Tosches and Laurent, 2019; Yamashita et al., 2018). Beyond those classical markers, single cell RNA-sequencing has provided transcriptomic evidence of the existence of the broad cortical pyramidal neurons in reptiles (Tosches et al., 2018). Although some cortical layer markers were co-expressed by the same cells of the turtle dorsal cortex, broad upper-layer and deep-layer types were identified according to transcriptome and efferent connection. These data support the potential existence of a pallium possessing the two main glutamatergic cell types in the last common ancestor of amniotes. We propose that a conserved developmental mechanism initiates the program of glutamatergic neuronal differentiation in all amniotes (Fig. 6).

**Fig. 7:**
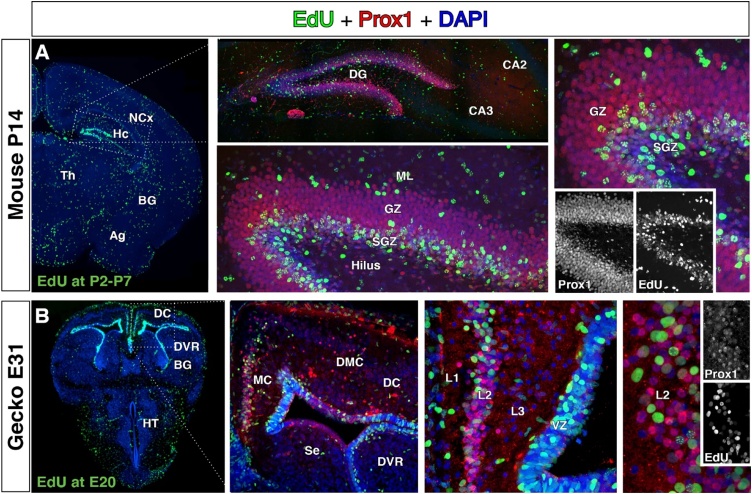
Conservation of dentate gyrus neuronal types in evolution. Coronal sections. A. The latest-born telencephalic neurons in the mouse brain were labelled with several administrations of the thymidine analogue EdU (from postnatal day P2 to P7, method described in (Sierra et al., 2015)). At P14, newborn neurons in the telencephalon (EdU+, green) were exclusively located in the dentate gyrus of the hippocampus, and typically expressed the granule neuron marker Prox1 (red). B. In the gecko *Paroedura pictus*, a single administration of EdU at the latest neurogenic period (embryonic day E20) labelled homologous dentate gyrus neurons (method as in (Nomura et al., 2013)). At E31, the birthdated neurons of the telencephalon (EdU+, green) located exclusively the medial cortex (MC -known homologue of the dentate gyrus) and expressed Prox1 (red), as their mammalian counterparts did. DAPI is shown in blue for counterstain. Ag, amygdala; BG, basal ganglia; CA1-3, *Cornu Ammonis* hippocampal regions 1–3; DC, dorsal cortex; DG, dentate gyrus; DMC, dorso-medial cortex; DVR, dorsal ventricular ridge; GZ, granular zone, Hc, hippocampus; HT, hypothalamus; L1-3, cortical layers 1–3; ML, molecular layer; NCx, neocortex; Se, septum; SGZ, subgranular zone; Th, thalamus.

Similar equivalence of neuronal types has also been found among pallial interneurons. Three main subtypes are identified in the pallium of mammals, chick, lizard and turtle regarding their expression of calcium-binding proteins such as somatostatin, parvalbumin or the serotonin receptor 5-HT3R (Reiner, 1993; Tosches et al., 2018; Tremblay et al., 2016). Although turtle *parvalbumin* type of interneurons do not express parvalbumin, transcriptomics shows that these interneurons express the whole set of markers common to mammalian parvalbumin-expressing interneurons. The general conservation of a broad plan for pallial neuronal types strongly suggests that these types were already present in the pallium of the last common ancestor. It also points to a hardcore developmental machinery required for broad specification, which might have not varied substantially in over 300 million years. Accordingly, we propose that the mechanisms for broad neuronal type specification are deeply conserved among amniotes. As an example, we show developmental, molecular and positional equivalence of hippocampal granule neurons in mammals and reptiles, a region of the pallium for which homology is not contested (Fig. 7). Our birthdating experiments show that Prox1 immunoreactive granule cells of the hippocampus share a developmental history both in mouse and gecko. These granule cells are in both cases the latest-born telencephalic cells during developmental neurogenesis, and are generated in the MPall of the two vertebrate lineages. The homology of these neuronal populations is supported by transcriptomic similarity (Tosches et al., 2018).

However, neuronal type specification moves beyond the main neuronal types, and dozens of neuronal subtypes can be described in some lineages. It was at this step when evolution tinkered and diversified pallial neurons further and beyond the conserved main types. As presented above, factors at both intrinsic genomic and extrinsic environmental levels played an evolutionary role. In the case of mammalian cortex, the number of different subtypes described for human, primates and mouse cortices increases as single cell-RNA technology improves. There are dozens of glutamatergic and GABAergic subtypes, each defined by their transcriptome, pattern of connectivity, location in the cortical area, and lamina (Hodge et al., 2019; Khrameeva et al., 2019; Polioudakis et al., 2019). Nothing as complex can be identified in reptiles. Although turtle pallial neurons resemble the broad mammalian types, these reptilian neurons do not differentiate further into the wide mammalian diversification (Tosches et al., 2018). At least one molecular mechanism comes to hand to explain the simpler variety of sauropsid neurons. Both bird and turtle pallia host neurons expressing a variety of combinatorial transcription factors code. But these species miss the Satb2-only expressing neurons, abundant in mammalian cortices (Nomura et al., 2018). The mammalian Ctip2 locus possesses an enhancer motif strongly suppressed by the transcription factor Satb2. This motif is absent in the sauropsid Ctip2 locus, which implies that Satb2 expression does not repress Ctip2 expression. This apparently minor genomic difference implies that sauropsid neurons do not reach the variety of combinatorial codes that mammalian neurons exhibit, and sauropsid neurons are therefore less diverse (Fig. 6). Moreover, heterochroninc changes in the regulation of these genes also underlie the efferent connectivity of each neuronal type. Marsupial cortical neurons express Satb2 earlier than the homologue neurons in mice. Due to this early expression, marsupial interhemispheric cortical neurons project via the anterior commissure instead of the corpus callosum as in eutherian mammals (Paolino et al., 2020).

For birds, the complexity of neuronal subtypes may rival that found in mammals, as suggested by some astonishing features of parrots and corvids: their neuronal numbers and densities are comparable to that of primate brains (Olkowicz et al., 2016), and their complex behavioral repertoires can only be achieved by complex, diverse neuronal networks. However, we still lack a thorough comparison of pallial neurons of birds, and the field awaits single cell RNA studies in birds equivalent to those of numerous investigations in mammals. However, single cell transcriptomic analysis shall have to be coupled to lineage analysis during embryonic development to establish developmental origin and draw conclusions on possible homologies.

Together with intrinsic differences, we must remember that the mantle zone where pallial neurons differentiate is widely diverse across amniotes, as a result of the diverging cumulative developmental programs depicted above. The cortical plate displays a distinct differentiation framework that has no molecular equivalence in other species (Tanaka et al., 2011). In summary, early development differences paved the road of pallial diversity, and this diversity was boosted by divergences in the fine cell differentiation programs.

## 7 Is it there a true neocortical homologue?

Following our view of homology as a product of a shared developmental programs, all dorsal pallial derivatives of amniotes are homologous as dorsal pallial derivatives, i.e., the neocortex in mammals, the dorsal cortex of reptiles, and the hyperpallium of birds. Although the final biological product of each of them is molecularly, cellularly, structurally, and functionally different, these differences may not obscure their true shared embryonic origin in the dorsal pallium, a unique sector of the pallium present in the last common ancestor to all and in all its descendant species. In a similar way as whale fins and human arms are homologous -regardless of their fine molecular and cellular composition and their divergent functions- the neocortex is homologue to the reptilian dorsal cortex and the avian hyperpallium, regardless of where these structures receive neuronal connections from and what their transcriptomic similarities are. The arm and fin are homologous as forelimb bud derivatives but not as either arms or fins. Arms and fins have homologous bones for the upper arm, forearm, and hand in a person and the respective bony components of a fin. So why not call a fin an arm or arms fins? It is because of the differences that subsequently emerged. This applies to pallial sector derivatives; the adult phenotypes are substantially different.

We invoke a study of homology strongly based on the developmental trajectory of organs and cells, which opposes to that of multileveled homology (Briscoe and Ragsdale, 2018). According to the latter view, brain homologies can be defined at several levels such as genetic, cellular, hodological, developmental, among others, being none of them more relevant than the others for the finding of shared traits. This view tends to forget that the so-called different levels of homology are not independent one another. The developmental history of a given cell explains all the other levels: it defines its genetic signature and mature transcriptome, which in turns is responsible for the functionality of the cell. In the specific case of neurons, it is known that every neuronal type is determined, defined and described solely by its developmental origin and path (Zeisel et al., 2018). This is why we encourage the study of brain development across vertebrate taxa, because comparative neurodevelopment is capable of revealing how brain evolution unfolds. We foresee that EvoDevo and neurobiology will embrace novel methodologies such as single cell RNA sequencing, viral mediated axonal tracing of genetic-specific populations and comparative epigenomics (Emera et al., 2016; Hu et al., 2017; Norimoto et al., 2020; Reilly et al., 2015; Tosches, 2017; Tosches et al., 2018), along with traditional neuroanatomy and experimental embryology. All of them will be key to unravel cortical evolution as well as other brain regions evolutionary history.

We have shown here the particular case of the mammalian neocortex. The developmental sector that produce this significant structure is well identified in mammals and its homologues are known across vertebrates. This sector, the dorsal pallium, evolved its divergent relevance due first to the varied power of the forebrain signaling centers. The differences in dorsal pallium size and the action of morphogens promoted the appearance of new precursor cell types. As a consequence, divergent and more populated germinative zones appeared across the amniote embryonic brains. Accompanied by novel cell populations, which arrived in the dorsal pallium by tangential migration from external sources, the dorsal pallium evolved to diversify the neuronal production. Ultimately, transcriptional signaling also evolved, enabling the differentiation of multiple neuronal subtypes in different taxa. All these evolutionary variations, together, enabled the variation of development that enabled the production and existence of the neocortex. These facts are known thanks to the comparison of brain development in a range of animal models. However, much more detailed lineage studies are required to map out the homology of brain structures. Lineage studies, such as those we performed on the tangentially migrating glutamatergic neurons and the ventral and lateral pallia are just the beginning of understanding this logic (Bruguier et al., 2020; García-Moreno et al., 2018; Rueda-Alaña et al., 2018). Comparative cell lineage combined with other methods, such as single RNA sequencing, novel viral-aided axonal tracing, and functional studies will be key in unravelling the evolutionary history of the brain. However, the comparative developmental perspective remains as the pivotal and essential framework.
