# Supplementary material for: Variations of telencephalic development that paved the way for neocortical evolution
Source: Prog Neurobiol. 2020 Nov;194:101865. doi: 10.1016/j.pneurobio.2020.101865 (PMC7656292; doi:10.1016/j.pneurobio.2020.101865)
Supplement: Supplementary file 1 [file mmc1.pdf]

Peer Review Overview

Manuscript Title: “Variations of telencephalic development that paved the way for neocortical evolution”

Received	28-Mar-2020
1st Decision	05-May-2020
Revision Submitted	29-May-2020
Accepted	04-Jun-2020

Decision Letter

Manuscript Number: PRONEU-D-20-00135
Title: Variations of telencephalic development that paved the way for neocortical evolution

Dear Zoltan,

Thank you for submitting your manuscript to Progress in Neurobiology.

We have completed our evaluation of your manuscript. The reviewers recommend reconsideration of your manuscript following major revision. We invite you to resubmit your manuscript after addressing the comments below. Please resubmit your revised manuscript by Jul 04, 2020.

When revising your manuscript, please consider all issues mentioned in the reviewers' comments carefully: please outline every change made in response to their comments and provide suitable rebuttals for any comments not addressed. Please note that your revised submission may need to be re-reviewed.

Progress in Neurobiology values your contribution and we look forward to receiving your revised manuscript.

Kind regards,

Sabine

Sabine Kastner, MD, PhD
Editor-in-Chief
Progress in Neurobiology

Editor and Reviewer comments:

Reviewer 1

The authors reviewed conserved and diversified developmental programs and cellular behaviors in corticogenesis, with special interests of amniotes, for the understanding of the

origin and evolutionary processes of mammalian neocortex. Clade-specific modifications in the dynamics of progenitor proliferation, differentiation, and migration, all of these could have great impacts on the final morphology of the pallial derivatives, and recent reports including the authors' elegant works shed lights on the part of underlying molecular mechanisms that provided species-specific cortical diversities. The article has been written in well and highly informative for general readers who are interested in neocortical development and evolution. I suggest a few minor revisions to further improve the quality of the article for the publication.

1. Figure 1. I understand the concept of the illustration that the balance of dorsal and ventral factors could contribute to species-specific pallial morphology; although sfrp1 is a fascinating molecule that attenuates canonical Wnt signaling, sfrp1 expression is not restricted in the ventral pallium but is detected broadly in the dorsal pallium (Miao et al. Front Mol Neurosci 2018 and elsewhere). Rather, sfrp2 (and Wnt7b) shows more restricted expression in the ventral pallium (or anti-hem), although there is no evidence how much these molecules actually contribute to the morphological diversity of the pallium.
2. A recent paper reported that the existence of claustrum and its role in slow-wave sleep in the reptiles (Norimoto et al. Science 2020), suggesting extensive conservations of ventral (or lateral) pallium-derived structures and its functions among amniotes. Also, the article provided some new directions on the comparative neurodevelopmental approaches combining physiology, comprehensive transcriptome analyses and adeno-associated virus-mediated circuit tracing. I suggest the authors include this article and discuss about the future directions on brain evo-devo research.
3. Some typos in the text, such as "But there I s another..).

Reviewer 2

This is a fine review/perspective piece. I only have a few suggestions for references and phrasing that are listed below:

REGARDING: “At the rostral region of the neural tube, the telencephalon gives rise to the sensory processing areas of all vertebrates, including the mammalian neocortex- and shows two major divisions: the subpallium (SPall) that generates mostly GABAergic neurons, and the pallium (Pall), which is the source of telencephalic glutamatergic neurons (De Carlos et al., 1996; Hevner et al., 2006; Tamamaki et al., 2003).”

Please reference:

Anderson, S., Mione, M., Yun, K. and Rubenstein, J.L.R. (1999) Differential origins of neocortical projection and local circuit neurons: role of Dlx genes in neocortical interneuronogenesis. Cerebral Cortex,9: 646-654.

REGARDING: “Based on conserved gene expression patterns of a few master developmental genes and transcription factors (Emx1, Pax6, Dlx1-2, Nkx2.1, among many others), the same subdivisions of both Pall and SPall areidentified in the embryos of all living amniotes (Puelles et al., 2000; Smith- Fernandez et al., 1998).”

These papers don't show data for fish - so be more cautious about saying "all living amniotes".

REGARDING: “Most likely, dorsal neural progenitors respond to patterning factors, namely Gli3, Shh, Wnt3a, Wnt2b, Fgf8, Fgf7, Sfrp1, Emx2, Pax6 or Ngn2 (Caronia-brown et al., 2014; Garda et al., 2002; Grove et al., 1998; Machold et al., 2003; O'Leary and Sahara, 2008; Rash and Grove, 2007;Yabut and Pleasure, 2018).”

Please reference:

Garel, S, Huffman, KJ, and Rubenstein, JLR. (2003). A caudal shift in neocortical patterning in a Fgf8 hypomorphic mouse mutant. Development. 130, 1903-1914.

Please reference the function of the Spry genes in cortical patterning: Faedo A, Borello U, Rubenstein JL. (2010). Repression of fgf signaling by sprouty1-2 regulates cortical patterning in two distinct regions and times. J Neurosci. 30(11):4015-23.

Please reference the function of CoupTf1: I suggest one of Michael Studer's papers.

Please reference the function of Pbx1:

Golonzhka O, Nord A, Tang PLF, Lindtner S, Ypsilanti AR, Ferretti E, Visel A, Selleri L, Rubenstein JLR. Pbx regulates patterning of the cerebral cortex in progenitors and postmitotic neurons. Neuron, 2015. 88(6):1192-207.

Please add a paragraph on the very likely role of the evolution of gene regulatory elements (i.e. enhancers, promoters) on controlling species specific expression patterns of critical patterning molecules in driving evolutionary modifications to cortical development. In this regard, you can illustrate the specificity of these regulatory elements to discrete embryonic telencephalic domains - a specificity that is rarely seen for gene expression, especially within the cortical primordium.

Reference:

Pattabiraman K, Golonzhka O, Lindtner S, Nord AS, Taher L, Hoch R, Silberberg SN, Zhang D, Chen B, Zeng H, Pennacchio L, Puelles L, Visel A, Rubenstein JLR. (2014). Transcriptional Regulation of Enhancers Active in Protodomains of the Developing Cerebral Cortex. Neuron. 82(5):989-1003.

REGARDING: “Thus, the same progenitor at late stages possess a restricted neurogenic potential, and will only produce the neurons typical of late neurogenic stages (Frantz and McConnell, 1996; McConnell, 1988).”

There is new evidence that this conclusion needs to be modified; please reference:

Temporal plasticity of apical progenitors in the developing mouse neocortex.

Oberst P, Fièvre S, Baumann N, Concetti C, Bartolini G, Jabaudon D.

Nature. 2019 Sep;573(7774):370-374. doi: 10.1038/s41586-019-1515-6.

Author Response Letter

Reviewer 1

The authors reviewed conserved and diversified developmental programs and cellular behaviors in corticogenesis, with special interests of amniotes, for the understanding of the origin and evolutionary processes of mammalian neocortex. Clade-specific modifications in the dynamics of progenitor proliferation, differentiation, and migration, all of these could have great impacts on the final morphology of the pallial derivatives, and recent reports including the authors' elegant works shed lights on the part of underlying molecular mechanisms that provided species-specific cortical diversities. The article has been written in well and highly

informative for general readers who are interested in neocortical development and evolution. I suggest a few minor revisions to further improve the quality of the article for the publication.

1. *Figure 1. I understand the concept of the illustration that the balance of dorsal and ventral factors could contribute to species-specific pallial morphology; although sfrp1 is a fascinating molecule that attenuates canonical Wnt signaling, sfrp1 expression is not restricted in the ventral pallium but is detected broadly in the dorsal pallium (Miao et al. Front Mol Neurosci 2018 and elsewhere). Rather, sfrp2 (and Wnt7b) shows more restricted expression in the ventral pallium (or anti-hem), although there is no evidence how much these molecules actually contribute to the morphological diversity of the pallium.*

Response: We agree with the reviewer that the contribution of Sfrp factors to telencephalic patterning is not well evidenced. We therefore only speculated that these could be well-suited candidates for the task, as they are secreted and could regulate Wnt signaling. We included Sfrp1 in the figure as it is the only of the two genes expressed in the avian ventral pallium (Frowein et al., 2002), but we understand that for mammals, Sfrp2 is a better candidate for our speculation. Accordingly, we have changed the mammalian part of the figure (Sfrp2 appears now), and have detailed the differences in Sfrp expression among amniotes in the text.

2. *A recent paper reported that the existence of claustrum and its role in slow-wave sleep in the reptiles (Norimoto et al. Science 2020), suggesting extensive conservations of ventral (or lateral) pallium-derived structures and its functions among amniotes. Also, the article provided some new directions on the comparative neurodevelopmental approaches combining physiology, comprehensive transcriptome analyses and adeno-associated virus-mediated circuit tracing. I suggest the authors include this article and discuss about the future directions on brain evo-devo research.*

Response: We thank the referee for this suggestion. We are aware of this influential new research piece and have included it now in the article. We first present it as additional support of the conserved radial development of the ventral and lateral pallia (page 13) and later as an example of how new methodologies will certainly solve the evolutionary history of brain regions and neuronal populations across vertebrates (page 21).

3. Some typos in the text, such as "But there I s another..).

Response: We have gone through the text carefully and corrected typos. We also asked a native speaker to read through the manuscript to eliminate typos.

Reviewer 2

This is a fine review/perspective piece. I only have a few suggestions for references and phrasing that are listed below:

1. *REGARDING: “At the rostral region of the neural tube, the telencephalon gives rise to the sensory processing areas of all vertebrates, including the mammalian neocortex- and shows two major divisions: the subpallium (SPall) that generates mostly GABAergic neurons, and the pallium (Pall), which is the source of telencephalic glutamatergic neurons (De Carlos et al., 1996; Hevner et al., 2006; Tamamaki et al., 2003).”*

Please reference: Anderson, S., Mione, M., Yun, K. and Rubenstein, J.L.R. (1999) Differential origins of neocortical projection and local circuit neurons: role of Dlx genes in neocortical interneuronogenesis. Cerebral Cortex,9: 646-654.

Response: We thank the referee for these suggestions. These references are now included.

2. *REGARDING: "Based on conserved gene expression patterns of a few master developmental genes and transcription factors (Emx1, Pax6, Dlx1-2, Nkx2.1, among many others), the same subdivisions of both Pall and SPall areidentified in the embryos of all living amniotes (Puelles et al., 2000; Smith- Fernandez et al., 1998)."*

   These papers don't show data for fish - so be more cautious about saying "all living amniotes".

Response: We are sorry to disagree about this particular comment by the reviewer. Fish are not amniotes. The articles we referenced (Puelles et al., 2000; Smith- Fernandez et al., 1998) show evidence of expression on birds, reptiles and mammals, the clades that comprise the amniote group (https://www.britannica.com/animal/Amniota).

3. *REGARDING: "Most likely, dorsal neural progenitors respond to patterning factors, namely Gli3, Shh, Wnt3a, Wnt2b, Fgf8, Fgf7, Sfrp1, Emx2, Pax6 or Ngn2 (Caronia-brown et al., 2014; Garda et al., 2002; Grove et al., 1998; Machold et al., 2003; O'Leary and Sahara, 2008; Rash and Grove, 2007;Yabut and Pleasure, 2018)."*

   - Please reference Garel, S, Huffman, KJ, and Rubenstein, JLR. (2003). A caudal shift in neocortical patterning in a Fgf8 hypomorphic mouse mutant. Development. 130, 1903-1914.

   - Please reference the function of the Spry genes in cortical patterning: Faedo A, Borello U, Rubenstein JL. (2010). Repression of fgf signaling by sprouty1-2 regulates cortical patterning in two distinct regions and times. J Neurosci. 30(11):4015-23.

   - Please reference the function of CoupTf1: I suggest one of Michael Studer's papers.

   - Please reference the function of Pbx1: Golonzhka O, Nord A, Tang PLF, Lindtner S, Ypsilanti AR, Ferretti E, Visel A, Selleri L, Rubenstein JLR. Pbx regulates patterning of the cerebral cortex in progenitors and postmitotic neurons. Neuron, 2015. 88(6):1192-207.

Response: We thank the referee for these excellent suggestions. All these references are included now and widen the catalogue of molecules related to telencephalic patterning.

4. *Please add a paragraph on the very likely role of the evolution of gene regulatory elements (i.e. enhancers, promoters) on controlling species-specific expression patterns of critical patterning molecules in driving evolutionary modifications to cortical development. In this regard, you can illustrate the specificity of these regulatory elements to discrete embryonic telencephalic domains - a specificity that is rarely seen for gene expression, especially within the cortical primordium.*

   Reference: Pattabiraman K, Golonzhka O, Lindtner S, Nord AS, Taher L, Hoch R, Silberberg SN, Zhang D, Chen B, Zeng H, Pennacchio L, Puelles L, Visel A, Rubenstein JLR. (2014). Transcriptional Regulation of Enhancers Active in Protodomains of the Developing Cerebral Cortex. Neuron. 82(5):989-1003.

Response: We thank the reviewer for this excellent suggestion, as we are strong supporters of a cell type and brain patterning evolution driven by regulatory mechanism changes, beyond coding sequences. We explain now in the article how evolution reached a variety of expression patterns of patterning factors thanks to changes of the regulatory mechanism of such genes. We also included the suggested reference.

5. *REGARDING: “Thus, the same progenitor at late stages possess a restricted neurogenic potential, and will only produce the neurons typical of late neurogenic stages (Frantz and McConnell, 1996; McConnell, 1988).”*

   There is new evidence that this conclusion needs to be modified; please reference:

   Temporal plasticity of apical progenitors in the developing mouse neocortex. Oberst P, Fièvre S, Baumann N, Concetti C, Bartolini G, Jabaudon D. Nature. 2019 Sep;573(7774):370-374. doi: 10.1038/s41586-019-1515-6.

Response: We thank the reviewer for pointing to this article. We referenced it in the article now, and we mention that these authors have challenged the concept of temporal restriction. However, this concept has shaped our understanding of cortical development over the last decades and perhaps this is premature to completely discard this concept based on the evidence provided by this particular study.
